# Edge level of aligners and periodontal health: a clinical perspective study in young patients

**DOI:** 10.1590/2177-6709.28.1.e2321124.oar

**Published:** 2023-04-14

**Authors:** Riccardo FAVERO, Lisa LIBRALATO, Francesca BALESTRO, Andrea VOLPATO, Lorenzo FAVERO

**Affiliations:** 1 Università degli Studi di Padova, Dipartimento di Neuroscienze (Padova/Veneto, Italy).

**Keywords:** Aligner, Fixed appliance, Early orthodontics, Periodontal, Esthetic

## Abstract

**Introduction:**

Although the superiority of clear aligners over multi-bracket appliances in keeping gingiva healthy has been suggested, the possible benefits of one aligner design over another have not yet been investigated, especially with regard to the vestibular edge.

**Objective:**

The aim of this study was to measure several periodontal indexes in adolescents undergoing orthodontic treatment with aligners, comparing two different types of rim.

**Methods:**

The study involved 43 patients aged between 14 and 18 years. The periodontal health was assessed using plaque index (PI), gingival index (GI), and gingival bleeding index (GBI), at the start of the treatment with aligners (T0), with a vestibular rim (VR) reaching up to 3 mm beyond the gingival margin. Three months later (T1), aligners were set to obtain a juxtagingival rim (JR) on the second quadrant and VR on the first quadrant. The periodontal indexes were measured again, both at T1 and then three months later (T2).

**Results:**

Intra-quadrant comparisons revealed a statistically significant worsening of the periodontal indexes only for the second quadrant (p<0.05), at T1 (GI), and especially at T2 (PI, GI, GBI), while no statistically significant changes were found for the first quadrant.

**Conclusions:**

More severe mechanical irritation, especially during insertion and removal of the aligner, can explain the worsening inflammatory indexes with the JR. In addition, the pressure exerted by the JR on the gingival sulcus seemed to facilitate plaque deposition, whereas the VR had a protective effect, reducing the risk of mechanical trauma.

## RESUMO

**Introdução:**

Embora se alegue uma superioridade dos alinhadores transparentes sobre os aparelhos multibraquetes em manter a gengiva saudável, ainda não foram investigados os possíveis benefícios de um modelo de alinhador sobre outro, especialmente no que diz respeito à borda vestibular.

**Objetivo:**

O objetivo deste estudo foi medir vários índices periodontais em adolescentes submetidos a tratamento ortodôntico com alinhadores, comparando dois tipos diferentes de borda.

**Métodos:**

O estudo envolveu 43 pacientes com idade entre 14 e 18 anos. A saúde periodontal foi avaliada por meio do índice de placa (IP), índice gengival (IG) e índice de sangramento gengival (ISG), ao início do tratamento com alinhadores (T0), com borda vestibular (BV) estendendo-se até 3 mm além da margem gengival. Três meses depois (T1), os alinhadores foram ajustados para se obter uma borda justagengival (BJ) no segundo quadrante e BV no primeiro quadrante. Os índices periodontais foram medidos novamente tanto em T1 quanto três meses depois (T2).

**Resultados:**

As comparações intraquadrantes revelaram uma piora estatisticamente significativa nos índices periodontais apenas para o segundo quadrante (p<0,05), em T1 (IG) e especialmente em T2 (IP, IG, ISG), enquanto nenhuma mudança estatisticamente significativa foi encontrada para o primeiro quadrante.

**Conclusões:**

Irritações mecânicas mais intensas, principalmente durante a inserção e remoção do alinhador, podem explicar a piora dos índices inflamatórios com a BJ. Além disso, a pressão exercida pela BJ no sulco gengival pareceu facilitar a deposição de placa, enquanto a BV teve um efeito protetor, reduzindo o risco de trauma mecânico.

## INTRODUCTION

Orthodontic treatment with aligners has attracted increasing interest in recent years and their use has become more widespread, coinciding with ongoing improvements in biomechanics and scientific evidence.^1^ ^,^ ^2^ In addition to their excellent aesthetic qualities and greater acceptance by patients, interest in this approach is also motivated by the opportunity to preserve periodontal health better than with the multi-bracket appliances, because aligners make dental hygiene easier to manage at home and at the dental office.^3^ Several studies^4^ ^,^ ^5^ compared the periodontal indexes, the total mass of the biofilm and the bacterial population, demonstrating the superiority of transparent aligners over fixed appliances in maintaining periodontal health. In a meta-analysis^6^ published in 2018, the authors suggested that, comparing with the traditional fixed appliances, patients treated with clear aligners have a better periodontal health; nevertheless, further investigations are recommended, since there are few randomized clinical studies and long-term evaluations.

Karkhanechi et al^7^ recently compared several inflammatory indexes (plaque index [PI], gingival index [GI], probing depth, and pocket depth) in adult patients with fixed multi-bracket appliances and aligners. Over the course of 12 months of orthodontic therapy, all the indexes were significantly lower in the patients with aligners. The authors concluded that aligners should even be recommended in the case of patients experiencing periodontal problems.

The potential toxicity for the gingiva and periodontal tissues of the plastics commonly used to produce aligners has recently been investigated.^8^ Despite the unavoidable dispersion of cytotoxic monomers in the juxtagingival environment, the results of the study showed that the effects were clinically irrelevant, given the negligible quantity of toxic molecules released.

No studies to date have considered the periodontal effects of aligners in adolescent patients, or how the two main types of rim used on aligners affect the patient’s periodontal health. The rim may be designed either to extend beyond the free gingiva line and reach into the vault on a level with the adherent gingiva, or to follow a juxtagingival course along each gingival outline. 

Thus, the aim of the present study was to identify any differences in periodontal indexes in relation to the type of rim on aligners used in a sample of adolescent patients.^9^ ^,^ ^10^ The null hypothesis was that the vestibular rim would not affect the adolescent patients’ periodontal health.

## MATERIAL AND METHODS

### SAMPLE SELECTION

This prospective observational study enrolled 48 patients. An informed consent was taken, according to a protocol approved by the Ethical Committee (No. 9n/AO/20). The inclusion criteria were: patients of both genders, self-sufficient in their oral hygiene procedures, undergoing orthodontic treatment with aligners at a dental clinic. Only Angle Class I patients with mild or medium crowding in the upper arch were considered, excluding severe crowding and complex cases that required additional biomechanical aids (power grips, inter-arch coils, etc). Only patients aged between 14 and 18 years were included. Further exclusion criteria included: evidence of dental caries, gingival or periodontal disease, tooth loss due to caries, and antibiotic treatments in the previous three months.

During the study, five patients were rejected due to poor compliance with the treatment recommendations. So the final sample consisted of 43 patients: 27 females (mean age 15.25 ± 1.65 years), and 16 males (mean age 15.65 ± 2.36 years). 

### PERIODONTAL INDEXES

Each patient attended a preliminary professional oral hygiene session 30 days before starting the treatment with aligners. During this study, each patient’s first and second quadrants were separately assessed three times, calculating the following periodontal indexes:

» Plaque Index (PI) (Loe & Silness, 1964), assessing the distovestibular, vestibular and mesiovestibular surfaces with a dental mirror, curette, and dry air jet. The final value was obtained from the sum of the PI obtained for each tooth divided by the number of teeth examined (Tables 1 and 2).

**Table 1:** Plaque Index (PI) scores.

Score 0	No plaque
Score 1	Thin film of microbial plaque along the free gingival margin
Score 2	Moderate plaque accumulation in the sulcus
Score 3	Large amount of plaque in sulcus or pocket along the free gingival margin

**Table 2:** Plaque Index (PI) interpretation.

0.1-0.9	Good plaque control
1.0-1.9	Adequate plaque control
2.0-3.0	Inadequate plaque control

» Gingival Index (GI) (Loe & Silness, 1963), assessing the distovestibular, vestibular and mesiovestibular surfaces with a periodontal probe. The tissue was dried, and the probe was applied to the outer surface to establish the gingiva’s consistency, then slid inside the sulcus to assess bleeding. The index was obtained by scoring each quadrant from 0 to 3 (Tables 3 and 4).

**Table 3:** Gingival Index (GI) scores.

Score 0	Normal gingiva: natural coral pink gingiva, with no inflammation
Score 1	Mild inflammation: slightly changes in color, slight edema. No bleeding on probing.
Score 2	Moderate inflammation: redness, edema and glazing. Bleeding upon probing.
Score 3	Severe inflammation: marked redness and edema/ulceration/tendency to bleed spontaneously

**Table 4:** Gingival Index (GI) interpretation.

0.1-1.0	Mild gingivitis
1.1-2.0	Moderate gingivitis
2.1-3.0	Severe gingivitis

» Gingival Bleeding Index (GBI) (Ainamo Bay, 1975), measured by sliding the probe into the sulcus, waiting 10 seconds and then identifying areas of bleeding. Three points were tested for each tooth, coinciding with the distovestibular, vestibular and mesiovestibular surfaces. A percentage was calculated from the number of sites showing bleeding divided by the number of sites examined, and multiplied by 100.

In this study, the ICCs were used for reliability testing, at a target value of 0.8 and a 95% CI of 0.2.

### ALIGNER

The aligners used in this study were made of 0.75-mm thick PET-G material by CA-Clear Aligner (Scheu Dental), and they were thermo-molded on resin models, previously scanned by an intraoral scanner^11^ (CS3600, Carestream, Rochester, NY, USA) programmed to obtain the necessary orthodontic movement.

### WORKFLOW

**Figure 1:**
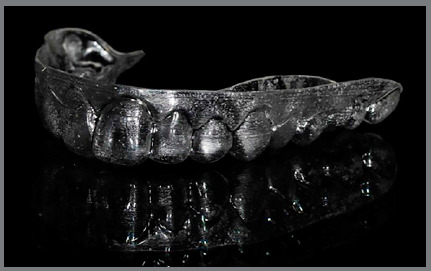
Aligner with vestibular rim on both sides, lateral view.

**Figure 2:**
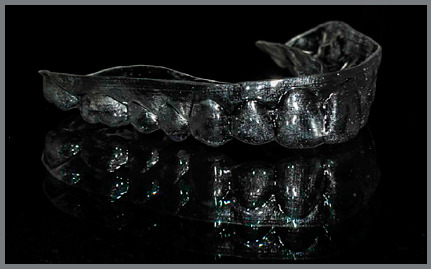
Aligner with vestibular rim on both sides, contralateral view.

**Figure 3:**
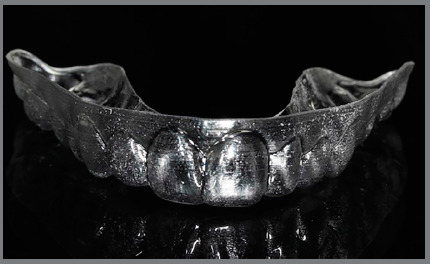
Aligner with vestibular rim on both sides, frontal view.

**Figure 4:**
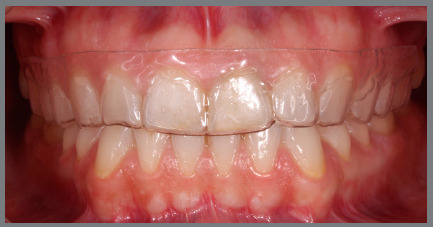
Aligner with vestibular rim on both sides, clinical view.

» At T0, 30 days after the session of professional oral hygiene, a periodontal health chart was completed for each patient, recording the three periodontal indexes separately for the first and second quadrants. Patients received the first aligners, with a vestibular rim (VR) extending approximately 3 mm beyond the gingiva line (Figs 1, 2, 3, and 4). 

**Figure 5:**
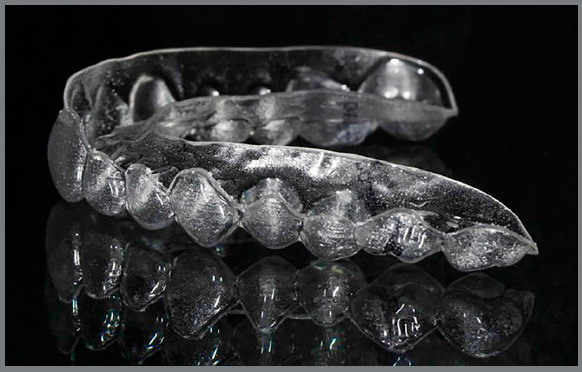
Aligner with vestibular rim on the first quadrant and juxtagingival rim on the second quadrant, lateral view.

**Figure 6:**
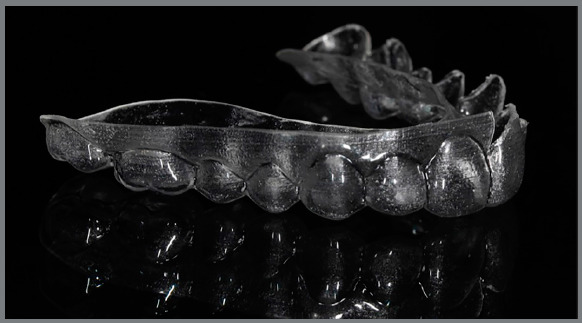
Aligner with vestibular rim on the first quadrant and juxtagingival rim on the second quadrant, contralateral view.

**Figure 7:**
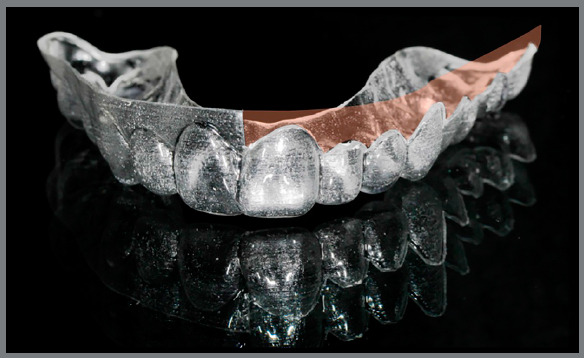
Aligner with vestibular rim on the first quadrant and juxtagingival rim on the second quadrant, frontal view.

**Figure 8:**
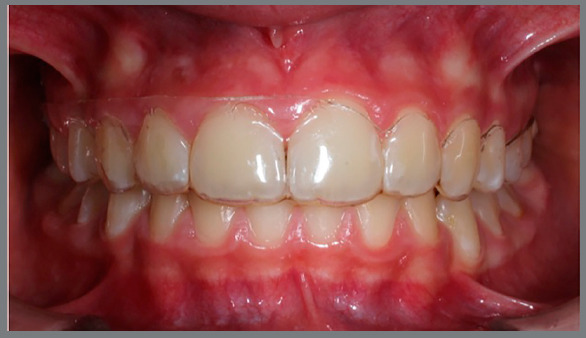
Aligner with vestibular rim on the first quadrant and iuxtagingival rim on the second quadrant, clinical view.

» At T1, three months later, the same periodontal indexes were measured, and patients underwent a session of professional oral hygiene. New aligners were delivered with a juxtagingival rim (JR) only for the second quadrant, shaped to suit the festooning profile of the papillae and gingival sulcus, and leaving the free gingiva uncovered (Figs 5, 6, 7, and 8).

» At T2, after further three months, the periodontal indexes were measured again and compared with the previous findings.

This procedure made it possible to identify differences in the periodontal health of the second quadrant, as compared with the first quadrant (which served as a control).

### SAMPLE SIZE

Supposing a 0.5 Cohen d standardized effect size in the intra-quadrant comparison, a sample size of at least 43 subjects would be enough to demonstrate if any difference exists in PI, GI, and GBI indexes. A *t*-test for paired sample was used, with a Type I error rate of 0.05 and Type II error rate of 0.20. The Bonferroni correction was used to the Type I error level for multiple endpoints.

### STATISTICAL ANALYSIS

The data are reported as medians (first and third quartiles) for continuous variables and percentages (absolute numbers) for qualitative variables. The Wilcoxon-Kruskal-Wallis test was used for continuous variables and Pearson’s chi-squared test for categorical variables. A Multivariate Marginal Model (MMM)^12^ was estimated, considering the results of all three Periodontal Indexes (PI, GI, and GBI). To take type I error rate inflation relating to multiplicity problems into account, adjustments for multiplicity were made using the Benjamini Hochberg procedure.^13^ The 95% CI was calculated for each value estimated. All computations were done using R software v. 3.3.2,^14^ with the mmm, gee, multcomp and RMS packages. 

## RESULTS

The results are summarized in Tables 5, 6, 7, and 8. In the intra-quadrant comparison, considering the first quadrant alone and comparing the trend of the three periodontal indexes over time (T0, T1, T2), no statistically significant results emerged for any of the indexes.

**Table 5:** Intra-quadrant comparison (first quadrant).

First quadrant	T0-T1 (P-value)	T1-T2 (P-value)	T0-T2
PI	0.135	0.59	0.056
GI	0.42	0.396	0.119
GBI	0.412	0.893	0.517

PI = plaque index, GI = gingival index, and GBI = gingival bleeding index.

*= statistically significant.

**Table 6:** Intra-quadrant comparison (second quadrant).

Second quadrant	T0-T1 (P-value)	T1-T2 (P-value)	T0-T2
PI	0.367	0.13	0.011*
GI	0.857	0.022*	0.03*
GBI	0.458	0.088	0.014*

PI = plaque index, GI = gingival index, and GBI = gingival bleeding index.

*= statistically significant.

**Table 7:** Inter-quadrant comparison.

First-Second quadrant	T0	T1	T2
PI	0.976	0.648	0.477
GI	0.18	0.359	0.025*
GBI	0.33	0.416	0.016*

PI = plaque index, GI = gingival index, and GBI = gingival bleeding index.

*= statistically significant.

**Table 8:** Multivariate marginal model; Model coefficients (95% Confidence Interval).

	mmmest	mmmIb	mmmub	pvmmm
PI time	0.08	0.02	0.14	0.01*
PI 1^st^ vs 2^nd^	0.01	-0.04	0.07	0.63
GI time	0.10	0.05	0.15	0*
GI 1^st^ vs 2^nd^	0.15	0.09	0.20	0*
GBI time	3.63	0.91	6.36	0.01*
GBI 1^st^ vs 2^nd^	4.33	2.20	4.66	0*

PI = plaque index, GI = gingival index, and GBI = gingival bleeding index.

mmmest = multivariate marginal model estimate, mmmlb= multivariate marginal model lower bound, mmmub= multivariate marginal model upper bound, pvmmm= p-value multivariate marginal model.

For the second quadrant, on the other hand, the results showed a statistically significant difference: GI showed significant worsening from T1 to T2 (P=0.022), as well as all three indexes from T0 to T2 (PI: p=0.01, GI: p= 0.03, GBI: p=0.014), following the aligner rim modification from VR to JR at T1.

Statistically significant differences also emerged from T0 to T2 in the inter-quadrant comparison, here again concerning the GI (p=0.025) and GBI (p=0.016) aggravation after the rim modification.

## DISCUSSION

This study investigated the effect of differently-shaped aligner rims on periodontal health and plaque formation. To date, there are no studies in literature aimed at investigating the effect of the flange of an aligner. Two edge designs are mainly available on the market: aligners with flange that extends into the vestibule for 3-4 mm and aligners with juxtagingival flange that follows the course of the tooth neck and the gingival sulcus. We therefore considered interesting to investigate the periodontal effect of these two main types of design. It was also decided to restrict the choice of the sample to adolescents due to the psycho-social peculiarities related to this particular phase of life, also in relation to habits and critical issues related to in-home oral hygiene.

Our patients’ periodontal health was examined and compared over time using indexes^15^ (PI, GI and GBI) already described in a meta-analysis^16^ comparing multi-bracket appliances with aligners.

Intra-patient comparisons were drawn using the same aligner with two different types of rim for patient’s two quadrants, to avoid influence by variations in patients’ dental hygiene routines at home. The first quadrant served as a “control” in order to monitor the trend of each patient’s dental hygiene throughout the trial. In the second quadrant, the aligner was used with the VR for the first three months, and with the JR for another three months. This approach avoided the risk of comparing quadrants associated with different levels of personal dental hygiene due to the influence of a patient’s dominant hand.

For the first quadrant (control), where the aligner rim remained the same, there were no statistically significant changes in the periodontal indexes at T1 or T2 - in other words, the patient’s dental hygiene did not change over the study period.

For the second quadrant, on the other hand, no changes emerged in the periodontal indexes from T0 to T1, when using the aligner with the VR, suggesting that the aligner had no influence on the patient’s dental hygiene. From T1 to T2, however, there was a statistically significant deterioration in the GI (p=0.022), attributable to the JR causing more severe trauma on a level with the gingival sulcus, with the tissues suffering mechanical irritation.

The comparison for the second quadrant between T0 and T2 generated the most significant data, with all periodontal indexes worsening to a statistically significant degree. There was also evidence of a statistically significant worsening of GI and GBI in the second quadrant by comparison with the first quadrant (p=0.025 and p=0.016). A deeper flange in the vestibule therefore appears more protective towards soft tissue than a flange adhering to the gingival sulcus.

Aligners with a JR presumably cause more trauma during their insertion and removal, giving rise to more inflammation on a level with the gingival sulcus. Because of the rim’s position along the gingiva line, it probably tends to push plaque inside the sulcus, and this would explain why the PI only increased in the second quadrant.

In contrast, the higher edge of the VR, positioned farther from the gingival sulcus, would provide better protection, causing less inflammation and a lower accumulation of plaque. Thus, the null hypothesis was rejected.

Despite an obvious limitation linked to a medium-low sample size, the choice for adolescents is interesting because this group is poorly investigated by clinical studies of transparent aligners. Further studies in other age groups are desirable to confirm the findings.

## CONCLUSIONS

» The use of aligners with a VR does not substantially affect the periodontal indexes in adolescent patients over time (p>0.05).
» In this population of young patients, aligners with a JR can lead to significantly worse periodontal indexes in the short term, and especially in the longer term (p<0.05).
» The mechanical irritation due to the aligner’s insertion and removal, and the effect of a JR in driving plaque inside the gingival sulcus may explain these different outcomes.
